# Smartphone-Based Light Detection and Ranging for Remote Patient Evaluation and Monitoring

**DOI:** 10.7759/cureus.16886

**Published:** 2021-08-04

**Authors:** Archis R Bhandarkar, Shaan Bhandarkar, Ryan M Jarrah, David Rosenman, Mohamad Bydon

**Affiliations:** 1 Neurosurgery, Mayo Clinic Alix School of Medicine, Rochester, USA; 2 College of Science, Yale University, New Haven, USA; 3 Neurosurgery, Mayo Clinic, Rochester, USA; 4 College of Arts and Sciences, University of Michigan-Flint, Flint, USA; 5 Internal Medicine, Mayo Clinic, Rochester, USA

**Keywords:** lidar, light detection and ranging, remote patient monitoring, telemedicine, smartphone

## Abstract

LIDAR (from “light detection and ranging” or “laser imaging, detection, and ranging”) is an evolving three-dimensional scanning technology with historical applications in various fields. However, the applicability of LIDAR in the field of medicine has mostly not been examined thus far. Here, we review the basic principles governing LIDAR and its potential to be used in three notable use cases in the context of remote patient monitoring: geriatric fall prevention, postoperative recovery monitoring, and home safety assessment. For assisting geriatric populations, LIDAR can create 3D renderings of their home environments and classify which objects may be associated with risk for falls. These risk factors can then be forwarded to both patients and providers in order for them to discuss how to make the patient’s environment safer. LIDAR is also capable of mapping the range of extremity motion in patients undergoing postoperative recovery. Such LIDAR data is simple to acquire and record for these patients and could enable unique metrics to be developed to assess patient outcomes in postoperative recovery. Finally, LIDAR can also reproduce 3D home models to identify attributes of their environments that could be harmful to infants. Given the recent momentum in telehealth following the events of the coronavirus 2019 disease (COVID-19) pandemic, LIDAR may also be a powerful tool in driving new insights from quality improvement initiatives through remote patient monitoring.

## Introduction

LIDAR (from “light detection and ranging” or “laser imaging, detection, and ranging”) is a form of three-dimensional scanning first developed in the early 1960s and soon used in meteorological, military, and aerospace settings [1]. As the technology has evolved, it has also become increasingly accessible and affordable. In late 2020, Apple announced that the iPhone 12 Pro (Apple Inc., Cupertino, California) would be equipped with LIDAR sensors, offering users the ability to three-dimensionally scan and map their own physical environments. While Apple’s intended use of LIDAR is to extend its smartphone’s augmented reality capabilities, the technology also opens the door to many new technological solutions that are of general interest to healthcare providers. In particular, LIDAR has great potential to augment existing remote patient monitoring solutions for a variety of patient-facing contexts, including evaluations of fall-risk in the elderly, physical rehabilitation following surgery, and crib safety for newborns. In this commentary, we summarize the basic principles and emerging use cases for mobile LIDAR, so that researchers and healthcare providers alike can be better equipped to integrate this technology with quality improvement initiatives going forward.

## Technical report

Basic principles of LIDAR

**Figure 1:**
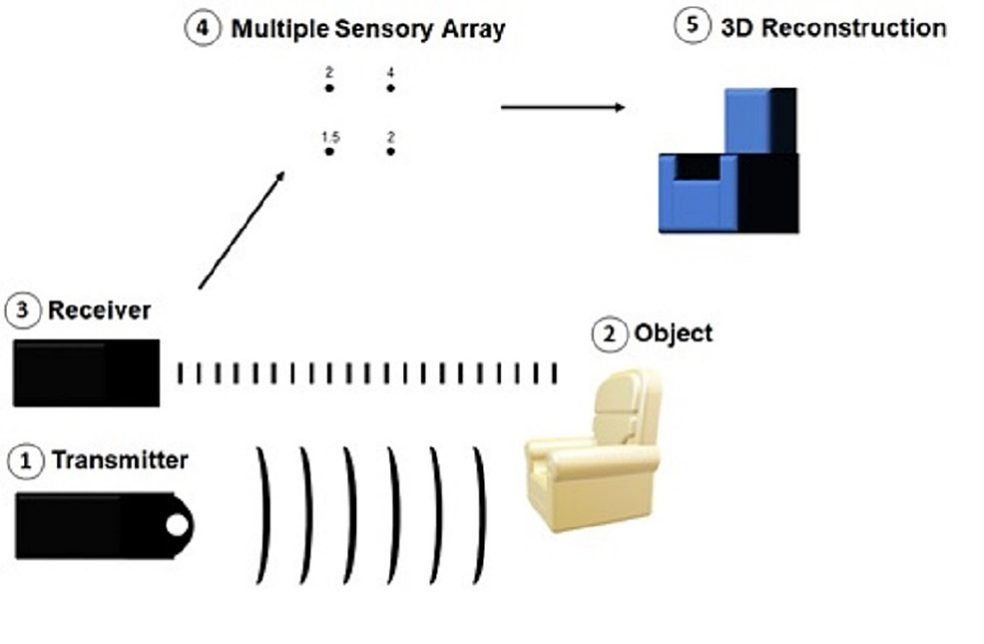
Schematic of the principles behind LIDAR technology

LIDAR sensors in smartphones can generate reliable three-dimensional portraits of a physical space through information gathered from infrared light pulses [2]. The basic steps of how LIDAR sensor data is generated include the following: (1) an array of transmitters sends pulses of infrared light into an environment, (2) objects in the environment reflect infrared light back, (3) an array of receivers measures reflected light, (4) a basic digital representation of the environment is created using differences in pulse travel time, and (5) a three-dimensional construction is generated based on the principle that farther away objects have longer pulse travel times compared to closer objects (Figure 1)**.**

Use Case 1: fall prevention in geriatric populations

As the United States population continues to age, falls in the elderly have become a growing public health concern that can result in significant morbidity and hospital costs for patients who suffer fall-related fractures. Many quality improvement programs have been launched to reduce at-home and in-hospital falls in the elderly. There is evidence demonstrating that multifactorial fall-prevention strategies, which combine exercise, vision assessment, and environmental modification can indeed lower the risk of injurious falls in the elderly [3-4]. The strategy of environmental modification focuses on removing or replacing fall risk factors from patients’ homes, including staircases without rails, slippery surfaces, worn-out carpets, and others [5]. Home assessments for fall risk factors have traditionally been performed through in-person visits from either physicians or allied health staff; however, this can be both resource and time-intensive when attempting to scale up a fall prevention program to a large cohort of patients. The dependence of these strategies on in-person visits is also a fundamental limitation, especially in retrospect of quarantines during the coronavirus disease 2019 (COVID-19) pandemic and the limitations that lockdown imposed on physical activity in patients.

Smartphone-based LIDAR provides an intuitive, remote patient monitoring solution that can meet the need for a scalable platform for assessing fall risk at home. Patients can use mobile applications on their smartphones like Scaniverse to create three-dimensional renderings of their homes, which can be shared with their healthcare providers [6]. Both manual and automatic computer vision-based annotation of objects in patient homes can be used to easily identify fall risks. The technology is scalable in that it reduces the need for providers to visit multiple patients’ homes. Moreover, the annotated three-dimensional renderings of patient homes can easily be shared with caregivers virtually so that they can be notified of fall risks. While companies like the United Kingdom-based Cera Care have used LIDAR for this particular use case, new smartphone-based LIDAR can expand public access to this technology and make it a readily available tool to augment fall prevention programs [7].

Use Case 2: monitoring postoperative recovery

Many quality improvement programs focusing on improving postoperative recovery workflows require quantifiable measures of patient health after returning home from the hospital. Several mobile technology-based solutions already exist for remotely quantifying patient outcomes after surgery. For example, one study used Fitbits to monitor step counts after cancer surgery in order to predict hospital readmission [8]. However, wearable devices like Fitbits can only provide crude data like step counts and require significant compliance from patients in order to wear the sensors throughout daily life.

Smartphone-based LIDAR can overcome these technical challenges and gather outcomes from patients in a non-intrusive way. Applications like Complete Anatomy have implemented LIDAR to three-dimensionally scan patients and calculate the range of motion in all extremities [9]. Such smartphone applications can be used to track recovery in the range of motion after surgery. Moreover, this use case of LIDAR is non-intrusive in that patients need only to submit videos of themselves after surgery, rather than having to wear a device for an extended period of time.

Use Case 3: home safety for infants and toddlers

Each year, approximately 2,300 infants die suddenly during sleep [10]. Several characteristics of the physical environment have been identified to increase the risk of such deaths. These include sleeping on soft surfaces or with soft objects or bedding, co-sleeping with others, and overheating, among others [11]. Approaches to assessing and risk-mitigating the sleeping environment have included home visits and the use of brochures and children’s books [12]. A three-dimensional reproduction of the sleeping environment could help healthcare providers understand the infant’s environmental context in a more detailed manner than photography or videography alone, possibly obviating the need in some cases for a home visit.

Likewise, toddlers can be at particularly high risk for injury in unsafe home environments. Risks of falling downstairs, off of a changing table, out a window; ingestion-related poisoning or choking; and contact-related burning or scalding all may be assessed in part by low- (or no-) cost, easily obtainable, and readily shareable three-dimensional images of the home environment.

## Discussion

Through the use cases mentioned above among other theorized uses in biomedical imaging, LIDAR bears immense potential in how it can be applied to improve patient care. Owing to its accessibility, LIDAR will produce a consistent stream of data that can be offered to drive quality improvement initiatives even at different levels of care. For example, emergency medical technicians (EMTs) are critical to linking geriatric patients with local fall prevention programs based on data from previous fall victims. Yet, the on-scene intervention information EMTs provide for falls is mostly derived from 9-1-1 reports and phone interviews with victims, with only minimal information about the patient’s actual spatial environment [13]. Remote monitoring with LIDAR can guide patients in deciding how to physically modify their home environments to remove risk factors. Geriatric patients can especially authorize other family members or caretakers to remotely access this data and assist them in making these alterations. Personalized instructions based on analysis of LIDAR data may then direct follow-up discussions between providers and patients. Cross-trained firefighters can also access this data to provide information about objects or structures that could become fire hazards. In fact, aerial LIDAR has been used in the past to assess structures in forests that present wildfire risks [14]. Hospital staff may also use the same LIDAR data to monitor patient movement after certain procedures or for those who are deemed high-risk for repeated falls. The widespread use of LIDAR can thus enable effective care coordination between public safety and health care systems.

Amidst quarantines and social distancing measures, the COVID-19 pandemic has illuminated the need for the integration of digital health technologies to improve patient outcomes. LIDAR is a remarkable technology that can capitalize on this movement in patient care and extend the reach of the health system. As described in the use case about patients undergoing postoperative recovery, LIDAR allows for remote evaluation by providers via simple recordings of patient movement. Other patients with mobility or even transportation issues would benefit greatly from the flexibility of a remote monitoring option for health evaluations. This would expand access to care for patients that may be in rural or other remote areas with limited numbers of physicians. Aside from improving the accessibility of health care, LIDAR may soon evolve into a diagnostic tool too. In some experiments involving patients with high alcohol intake or movement disorders like Parkinson’s, LIDAR has also been employed to quantify gait or other movement abnormalities by measuring oscillations in said movement [15]. As the technology becomes more popular with its introduction into the Apple iPhone 12 Pro, LIDAR has the potential to be a staple of the new wave of telehealth.

Despite the versatility of LIDAR’s applications, there may be some limitations to the technology’s widespread implementation that may be worth considering. As with any remote patient monitoring solution, long-term collection of data may conflict with patient privacy concerns. Patients may not wish their every action or movement to be recorded regardless of the reason. The LIDAR data should also be robustly secured so as not to leak critical information about the patient’s physical surroundings or potentially protected health information. Most importantly, LIDAR can result in large datasets that will require a high level of analysis and interpretation by specialists. However, the hope is that as LIDAR use becomes normalized in the field of medicine and beyond, proper safety measures and data interpretation pipelines can be instituted to address these shortcomings and magnify LIDAR’s benefits.

## Conclusions

New smartphone-based LIDAR is an innovative solution for remote patient monitoring that can enhance data collection across several patient contexts. As more users adopt devices like the iPhone 12 Pro and its counterparts, LIDAR sensing will be able to be integrated into a variety of quality improvement programs settings including fall prevention and post-operative recovery. We believe that the principles and use cases laid out in this article will help stimulate further applications of this emerging technology to improve patient outcomes.
